# Fault Diagnosis Method for Industrial Robots Based on DBN Joint Information Fusion Technology

**DOI:** 10.1155/2022/4340817

**Published:** 2022-03-26

**Authors:** Jian Jiao, Xue-jiao Zheng

**Affiliations:** Chongqing Water Resources and Electric Engineering College, Chongqing 402160, China

## Abstract

Aiming at the problems of the traditional industrial robot fault diagnosis model, such as low accuracy, low efficiency, poor stability, and real-time performance in multi-fault state diagnosis, a fault diagnosis method based on DBN joint information fusion technology is proposed. By studying the information processing method and the deep learning theory, this paper takes the fault of the joint bearing of the industrial robot as the research object. It adopts the technique of combining the deep belief network (DBN) and wavelet energy entropy, and the fault diagnosis of industrial robot is studied. The wavelet transform is used to denoise, decompose, and reconstruct the vibration signal of the joint bearing of the industrial robot. The normalized eigenvector of the reconstructed energy entropy is established, and the normalized eigenvector is used as the input of the DBN. The improved D-S evidence theory is used to solve the problem of fusion of high conflict evidence to improve the fault model's recognition accuracy. Finally, the feasibility of the model is verified by collecting the fault sample data and creating the category sample label. The experiment shows that the fault diagnosis method designed can complete the fault diagnosis of industrial robot well, and the accuracy of the test set is 97.96%. Compared with the traditional fault diagnosis model, the method is improved obviously, and the stability of the model is good; the utility model has the advantages of short time and high diagnosis efficiency and is suitable for the diagnosis work under the condition of coexisting multiple faults. The reliability of this method in the fault diagnosis of the joint bearing of industrial robot is verified.

## 1. Introduction

Currently, automated production is developing rapidly in the direction of automation and intelligence. More and more companies use industrial robots to replace traditional manual operations, which significantly improves production efficiency while reducing labour costs [1, 2]. In automated production operations, the division of labour among the robots in each segment is relatively straightforward, and the synergistic relationship is close. When a robot malfunction occurs, it will inevitably lead to problems in the whole operation process, which has a significant impact on the progress and safety of the operation [3–5]. Therefore, it is necessary to judge the operation status of industrial robots in advance and deal with the initial failure in time to avoid various safety hazards caused by robot failure.

Based on this, some scholars used different methods to complete the fault diagnosis of industrial robots. Verma and Simmons [6] established a plan of robot fault diagnosis based on discrete-time observers by designing observers. The method achieves the fault diagnosis of robot joints through the cooperation of detection and diagnosis observers, which requires a large amount of joint sensor information. Jaber and Bicker [7] collected the vibration signal of the working state of the robot, used the methods of wavelet transformation time-frequency domain analysis to analyze the fault signal of the robot under various working conditions, and realized the fault diagnosis of the robot. Capisani et al. [8] established a fault diagnosis model for robots using a sliding observer and applied it to the fault diagnosis of Comau robots. This method can accurately diagnose a single fault, but it does not perform well in diagnosing robots with multiple responsibilities. Long et al. [9] used sparse hybrid autoencoder (SAE) and support vector machine (SVM) to build a basic fault diagnosis model by learning fault information posture dataset, which can better complete the diagnosis of different faults of multi-joint industrial robots. However, it is difficult to implement large-scale sample training, and the accuracy of multi-fault classification is not high. Shan et al. [10] presented a fault diagnosis method for rolling bearings based on variable mode decomposition (VMD) and backpropagation (BP) neural networks. The energy of each component is obtained by decomposing the time-domain signal of bearing vibration into an intrinsic mode function, and the point is input as a feature into the training of the BP network. A diagnostic model for VMD-BP is established. The bearing fault type diagnosis can be completed well, but the accuracy of diagnosis is not ideal due to the boundary effect and the impact of sudden signals.

With the deepening of fault diagnosis research, the fault diagnosis method based on DBN has achieved good results in mechanical fault diagnosis such as bearing, gearbox, and motor [11–13]. Therefore, taking the joint bearing of industrial robot as the research object and the vibration signal as the starting point, this paper constructs the fault diagnosis method of industrial robot based on DBN. At the same time, considering that the accuracy of information fusion conclusion of traditional D-S theory is not ideal, the fusion problem of high conflict evidence is solved by improving D-S evidence theory. Taking the output layer of the model as fault evidence, the conflict between sample evidence is analyzed by using the improved D-S fusion rules and decision rules. Finally, a fault diagnosis model based on DBN joint improved D-S is established to improve the recognition accuracy of the fault model. It is of positive significance to further improve the service life and operation safety of industrial robots.

## 2. Related Knowledge

### 2.1. Information Fusion Technology

Information fusion technology is a multi-level and multi-faceted statistical process in its essence by detecting and combining the estimation of multiple sources of data to obtain information that information fusion can use [14]. Unlike simple signal processing techniques, multi-source information fusion techniques are suitable for handling multi-modal and conflicting forms of data and can achieve different levels and conditions of information fusion [15]. Thus, it performs well in improving the real time and reliability of mechanical systems, increasing the detectability of mechanical systems, and reducing the uncertainty of mechanical equipment.

**Figure 1:**
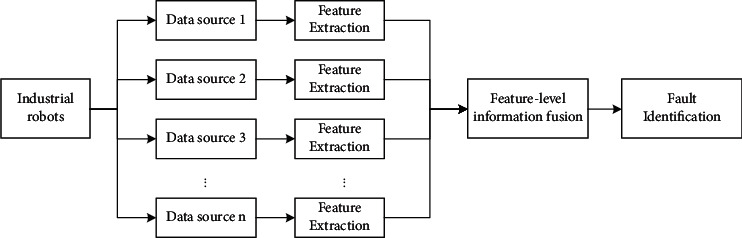
Feature layer fusion.

According to the different levels of fusion processing, information fusion is mainly divided into three groups: data layer fusion, feature layer fusion, and decision layer fusion. Among them, the feature layer fusion technology extracts the corresponding features of the sensor data based on the type of the original signal, fuses the resulting feature information in the feature layer, and after normalization, forms a single feature vector to finally complete the classification of information and realize the identification of faults. The fusion process is shown in Figure 1.

### 2.2. Deep Belief Networks

**Figure 2:**
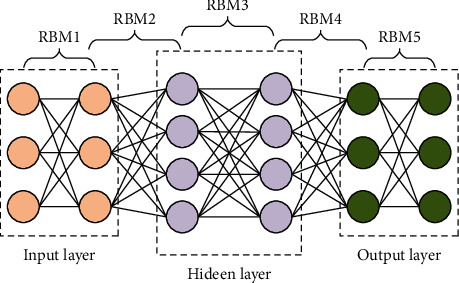
DBN structure.

Deep belief network (DBN) is a kind of neural network that can perform the tasks of feature recognition, data classification, and generation well. The scalability of the network is vital, so it is widely used in machine learning. DBN framework adopts the restricted Boltzmann vector machine (RBM) structure, and the model consists of visible, hidden, and output layers. In the DBN model, any two adjacent layers can be considered one RBM structure. The number of neurons in the visible layer is consistent with the dimension of the input data, which is mainly responsible for receiving the data from the bottom layer and outputting the computation results to the hidden layer. The BP artificial neural network (BPNN) in the top layer of the network classifies the features. It combines some of the labelling information to fine-tune the network parameters backwards to obtain the optimal network model [16, 17]. The DBN structure is shown in Figure 2.

**Figure 3:**
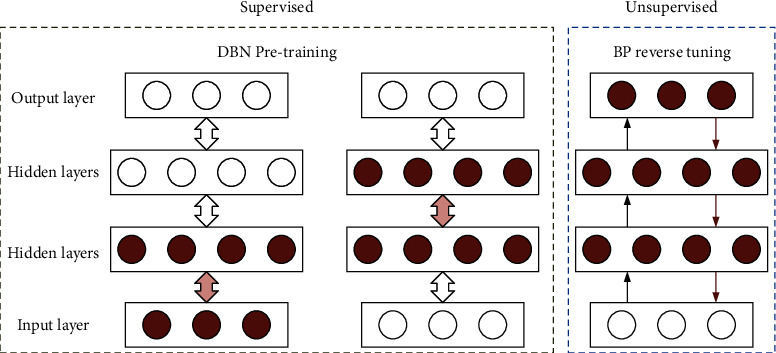
DBN training process.

The probabilistic generative model used in DBN differs from the discriminative model of traditional neural networks. DBN uses probability generation models to build the joint distribution of data and labels, train the hidden and visual layers of the network layer by layer, update and optimize the weights between layers and transfer parameters continuously so that the entire network model can generate training data with maximum probability, mine the correlation between higher-order data, and realize the extraction, classification, and identification of fault feature data [18]. The neurons in the same layer of the network are independent of each other and connected with the neurons in the adjacent layer, which makes the network have good conditional independence, which improves the parallel computing ability of the network and dramatically improves the training efficiency. Taking the DBN containing two RBMs as an example, the network training process is shown in Figure 3.

The training process of DBN consists of two main phases: unsupervised pretraining and supervised reverse fine-tuning.

1. *Unsupervised Pretraining*. Multiple stacked restricted Boltzmann machines form a deep Boltzmann machine, and the output of the previous RBM is the input of the next RBM. Two steps carry out the training process of each RBM: forward computation and reverse reconstruction, and the optimal parameters of each RBM are finally obtained after multiple iterations in different batches.
2. *Supervised Inverse Fine-Tuning*. After the pretraining, the RBM extracts the original data features, then classifies them using the top-level classifier, and finally fine-tunes all parameters top-down using the BP algorithm in combination with the label information to finally obtain the optimal model parameters.

## 3. Fault Diagnosis of Industrial Robot Based on DBN

### 3.1. Vibration Signal Preprocessing

**Figure 4:**
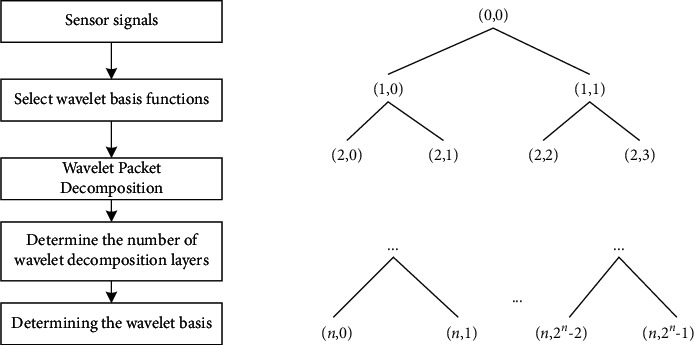
Wavelet transform flow and decomposition process.

The signal collected by the sensor contains the vibration of the joint itself and internal and external noise and non-smooth interference signals such as resonance signals, so the vibration signal needs to be processed for noise reduction for the next operation. Wavelet packet transform (WPT) can divide the signal in a multi-level form and decompose the high-frequency part in depth, adaptively select the frequency band according to the characteristics of the analyzed signal, ensure the frequency band and the signal spectrum match each other, and thus improve the time-frequency resolution [19]. Therefore, in this paper, wavelet packets are used to complete the noise reduction of industrial robot vibration signals. The wavelet transform process and decomposition process are shown in Figure 4.

The steps of noise reduction based on wavelet packet transform signal are as follows.

 
*Step 1*. Select the wavelet basis according to the decomposition level of wavelets and decompose the signal.
 
*Step 2*. Determine the optimal wavelet packet basis and calculate the optimal tree after the entropy criterion.
 
*Step 3*. Select an appropriate threshold value for the high-frequency coefficients at different decomposition scales and perform threshold quantization. The wavelet coefficients larger than the threshold value are considered to have signal generation and are retained; those smaller than the threshold value are deemed to be caused by noise and are set to zero, thus completing the purpose of noise reduction.
 
*Step 4*. Reconstruct the signal using the processed coefficients. The signal-to-noise ratio (SNR) and the root mean square error (RMSE) of the estimated signal and the original signal are often used to judge the merit of the denoising performance [20].

### 3.2. Energy Entropy Normalized Eigenvectors

After the vibration signal is processed by wavelet transform, it is not suitable as the underlying input to the DBN due to the vast amount of information contained in the signal [21]. The signal's energy entropy normalized feature vector is further calculated, and the energy entropy normalized feature vector is used as the underlying input to the DBN model.

The energy entropy represents the measure of uncertainty [22]. It is defined as follows: if there is a system *S* containing multiple events *S* = {*E*_*1*_, *E*_*2*_,…, *E*_*n*_} inside, each with a probability distribution *P* = {*p*_*1*_, *p*_*2*_,…, *p*_*n*_}, then the information of each event itself is

(1) $$\begin{array}{c} I_{e}=-\;\log_{2}p_{i}. \end{array}$$

The sum of the information entropy of all events in the whole system is the energy entropy, expressed as

(2) $$\begin{array}{c} E_{s}=\sum_{i=1}p_{i}I_{e}. \end{array}$$

After the wavelet transform of the vibration signal, a total of 2^*K*^ nodes are reconstructed, and the energy entropy of each node is calculated to obtain a feature vector composed of 2^*K*^ elements. The energy entropy function is expressed as

(3) $$\begin{array}{c} E\left(u_{K}\right)=-\sum_{J}u_{K}\log\left(u_{K}\right). \end{array}$$

The resulting energy entropy is normalized to [0, 1]. The energy entropy normalized eigenvector of the decomposed reconstructed signal is obtained as follows:

(4) $$\begin{array}{c} E\left(u_{J}\right)*=\frac{E\left(u_{J}\right)-E{\left(u_{J}\right)}_{\min}}{E{\left(u_{J}\right)}_{\max}-E{\left(u_{J}\right)}_{\min}}. \end{array}$$

### 3.3. Improving Information Fusion with D-S Evidence Theory

Dempster–Shafer (D-S) evidence theory is a kind of uncertainty inference, which effectively solves the influence of incomplete and uncertain information and other factors on the inference results by describing the uncertainty of the state of each part of the system from different perspectives and generalizing and estimating its probability. However, when the conflict between the evidence is severe, the traditional D-S theory information fusion accuracy is not satisfactory [23, 24]. To solve the fusion problem of high conflicting evidence, this paper uses the improved D-S evidence theory to complete the fusion of information to improve the identification accuracy of the fault model.

The inner vector product is added to the modification of the evidence combination rule. For any set of evidence, represented by a space vector, the average of each evidence vector is obtained by extending the evidence vector *w* to the *n* dimension as

(5) $$\begin{array}{c} \bar{w}=\frac{w_{1}+w_{2}+\cdots w_{n}}{n}. \end{array}$$

The distance between each evidence vector and the average evidence vector is

(6) $$\begin{array}{c} DM=\left[c_{1},c_{2},\ldots c_{n}\right],\\ c_{i}=\frac{\sqrt{2}}{2}c\left(w_{i},\bar{w}\right). \end{array}$$

The similarity measure *Sw*_*i*_=1 − *c*_*i*_ between two evidence vectors, i.e., the support of each evidence to evidence *i*, is calculated to represent the support of each evidence to evidence *Sup*(*w*_*i*_). Then, the weight *Crd*(*w*_*i*_) of evidence *w*_*i*_ is

(7) $$\begin{array}{c} \text{Crd}\left(w_{i}\right)=\frac{\text{Sup}\left(w_{i}\right)}{\sum_{i=1}^{n}\text{Sup}\left(w_{i}\right)}. \end{array}$$

By assigning a weighted average to the basic probabilities of each piece of evidence and then fusing the data, we can effectively solve the problem of conflicting evidence and further improve the accuracy of the fusion results.

## 4. Fault Diagnosis Model Based on DBN Joint Improved C-S Information Fusion

**Figure 5:**
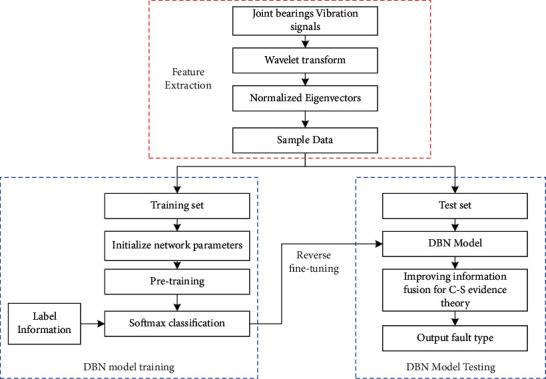
Industrial robot fault diagnosis model.

The DBN-based industrial robot fault diagnosis model is shown in Figure 5. The diagnosis model mainly contains the feature extraction part, DBN part, information fusion part, etc. Firstly, the vibration signal of the industrial robot joint is collected by the acceleration sensor. The wavelet packet transform is used to filter and reduce the signal's noise to avoid the deviation of the signal caused by the motion frequency and resonance frequency. The energy entropy normalized feature vector of the vibration signal is established using the information energy entropy theory. Secondly, the energy entropy normalized feature vector is divided into the training and test sets. The basic parameters of the DBN model are fine-tuned by unsupervised forward training and reverse supervised fine-tuning. The DBN model is constructed in this way. The output layer of the DBN model is used as the fault evidence for the evidence conflict factor analysis to check whether there is high conflict evidence, and the corresponding combination rules and decision rules are selected accordingly to realize the information fusion and complete the robot fault diagnosis.

## 5. Experimental Validation

### 5.1. Fault Dataset

The proposed method is used to construct a DBN classification model, and experiments are conducted to verify the effectiveness of the proposed method by acquiring vibration signals from the joint bearings of industrial robots. The experiments are shown on the Anaconda development platform running on the Windows 10 system as the base environment. The DBN deep learning model is built based on the Google deep learning framework TensorFlow.

Taking the FANUC Robot M-710C industrial robot as an example, set artificial fault on its joint bearing with *f* = 12.5 KHz frequency to collect collaborative vibration data. The data are divided according to the location and degree of the spot. Seven data types are obtained, including normal conditions and 350 sampled data points in each vibration cycle. Therefore, the original data of 7 categories are divided into 840 samples according to the window size, moving step of 1050 data points, and window moving step of 1050 data points to obtain the subseries sample space *R*^840×1050^, and each sample in the space is decomposed to calculate the time-frequency characteristics of each component to form a feature vector. According to the different fault categories corresponding to each feature vector, the category calibration is performed individually. The training and test sets are divided according to the ratio of 7 : 3.  Table 1 shows the industrial robot joint bearings' fault data and category labels.

In the experiment, wavelet transform is used to reduce the noise of all data signals. Then, the energy entropy normalized feature vector and fusion features of the signals are calculated. The features' characteristics are analyzed, and finally, the different feature sets are input to the DBN-CS model for verification. Then, the fault diagnosis of industrial robot joint bearings is completed.

### 5.2. Experimental Results

**Figure 6:**
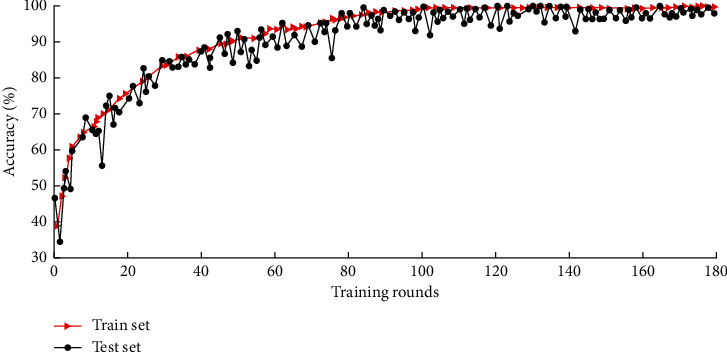
Accuracy variation curves of different sample sets.

Parameter setting of industrial robot fault diagnosis model is as follows: DBN network structure 128-100-36-7, network forward training is set to 100 times, and reverse optimization is set to 1000 times. The learning rate *ε* = 0.05, the momentum *m* = 0.6, the maximum number of iterations of the network is 50, and the training batch size is 100. Then, 180 rounds of training are carried out on the model using the training set, and the test set is used to verify the model's accuracy. The accuracy change curves of different sample sets are shown in Figure 6.

**Figure 7:**
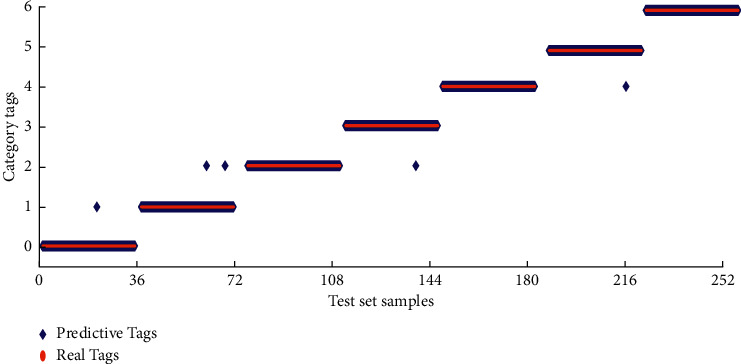
Comparison of predicted results and actual categories.

As shown in Figure 6, the accuracy rate increases with training rounds and stabilizes when the number of training rounds reaches 95. If we continue to train the data, it will not only not improve the accuracy significantly but also increase the computational time cost and even lead to the occurrence of overfitting. Therefore, the DBN model was tested after 95 rounds using the test set data, and the prediction results were compared with the actual category.  Figure 7 shows the comparison between the predicted results and the actual categories.

In Figure 7, if the category labels obtained by the classification model overlap with the corresponding actual category labels, the classification result is correct; otherwise, it means that the classification fails. Of the 252 test samples, 5 were misclassified (points offset in Figure 7). Among them, one fault-free state was misclassified as outer ring light fault, two external ring light faults were misclassified as outer ring medium faults, one outer ring serious fault was misclassified as outer ring medium faults, and one inner ring medium fault was misclassified as internal ring light faults. To quantitatively analyze the accuracy of different fault classifications, the confusion matrix is used to represent the classification results of DBN on the test set, and the test statistics are shown in Table 2. The rows and columns in the table indicate the actual fault type and the diagnosed fault type, respectively.

**Figure 8:**
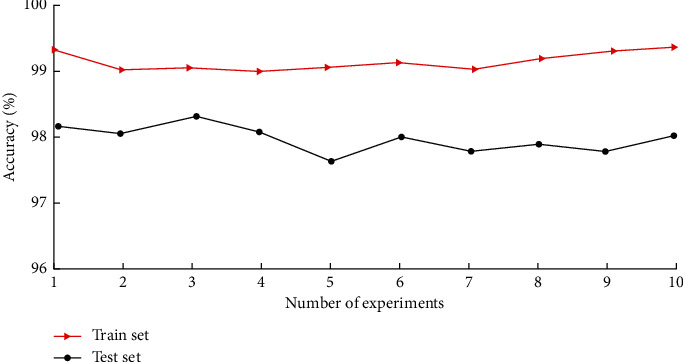
Accuracy of the experiment.

To verify the stability and adaptability of the diagnostic model in the paper, the model is trained ten times with the same parameters, and the data are selected from the dataset without repetition as the training set and the test set in a random way to test the detection accuracy of the fault diagnosis model. The classification results are shown in Figure 8.

As can be seen from Figure 8, the diagnostic accuracy of the training set varies smoothly, with an average accuracy of 99.12%; the accuracy of the test set is lower than that of the training set, but the average accuracy still reaches 97.96%, which shows that the model designed in the paper has a high fault diagnosis rate and good stability. It is suitable for the diagnosis of joint bearings in multi-state coexistence.

### 5.3. Performance Comparison of Different Diagnostic Models

To make the industrial robot fault diagnosis models designed in this paper comparable, different fault diagnosis models are constructed for testing with the same standard data, and the accuracy on the final test set is used as the evaluation criterion for model performance to verify the performance of the models in the paper. The fault diagnosis domain models involved are standard DBN, VMD + BPNN, VMD + SVM, EMD + DBN, and EMD + SVM models. The performance comparison results are shown in Table 3.

As can be seen from Table 3, compared with other algorithmic models, the diagnostic accuracy of the model designed in this paper is higher, and the standard deviation of the model is lower. Although the running time increases compared with VMD + SVM and EMD + SVM models, the increase is lower. It can better balance the relationship between diagnostic accuracy and real time to complete the fault diagnosis in complex fault states.

## 6. Conclusion

Taking industrial robot joint bearings as the research object, the problems of the current fault diagnosis methods are investigated by combining modern signal processing methods and deep learning theory. The accuracy rate is low when fault diagnosis is performed by the DBN alone, and it cannot meet real-time demand. Therefore, information fusion technology is combined to improve the performance of the fault diagnosis model to ensure the stability of the model and the accuracy of the diagnosis results. The experimental results show that the fault diagnosis accuracy of our method is higher than that of the traditional method and the average accuracy of the test set reaches 97.96%. The information fusion method with improved D-S evidence theory can effectively solve the evidence conflict problem and further improve the accuracy of the fusion results, suitable for handling the diagnosis of robot joint bearings under multiple fault states.

## Figures and Tables

**Table 1:** Joint bearing data and category labels.

Fault location	Degree of failure	Depth of failure (inch)	Training set samples	Training set samples	Failure tags
Fault-free	No	0	84	36	0

Outer ring	Minor	0.004	84	36	1
	Moderate	0.008	84	36	2
	Severe	0.012	84	36	3

Inner ring	Minor	0.004	84	36	4
	Moderate	0.008	84	36	5
	Severe	0.012	84	36	6

**Table 2:** Diagnostic accuracy of different faults.

Failure tags	0	1	2	3	4	5	6	Accuracy (%)	Average accuracy (%)
0	35	1	0	0	0	0	0	97.22	98.01
1	0	34	2	0	0	0	0	94.44	
2	0	0	36	0	0	0	0	100	
3	0	0	1	35	0	0	0	97.22	
4	0	0	0	0	36	0	0	100	
5	0	0	0	0	1	35	0	97.22	
6	0	0	0	0	0	0	36	100	

**Table 3:** Performance comparison of different fault diagnosis models.

Diagnostic models	Training set accuracy (%)	Test set accuracy (%)	Running time (s)	Standard deviation
DBN	87.34	81.66	61.63	0.0703
VMD + BP	92.72	86.58	39.17	0.0418
VMD + SVM	—	92.08	20.07	0.0658
EMD + DBN	90.21	86.17	48.55	0.0277
EMD + SVM	—	94.25	17.43	0.0491
The proposed method	99.12	97.96	23.46	0.0074

## Data Availability

The labelled dataset used to support the findings of this study is available from the corresponding author upon request.
